# *Bartonella* spp. seroprevalence in tick-exposed Swedish patients with persistent symptoms

**DOI:** 10.1186/s13071-021-05043-3

**Published:** 2021-10-12

**Authors:** Marie Edvinsson, Camilla Norlander, Kenneth Nilsson, Andreas Mårtensson, Elisabet Skoog, Björn Olsen

**Affiliations:** 1 Department of Medical Sciences, Section of Infectious Diseases, Uppsala University, Uppsala, Sweden; 2 Department of Medical Sciences, Section of Clinical Microbiology, Uppsala University, Uppsala, Sweden; 3 Department of Women’s and Children’s Health, International Maternal and Child Health (IMCH), Uppsala University, Uppsala, Sweden

**Keywords:** Bartonella, Ticks, Vector-borne disease

## Abstract

**Background:**

*Bartonella* spp. are emerging pathogens transmitted by arthropod vectors, possibly including ticks. We have investigated signs of bartonellosis in Swedish patients with presumed tick-bite exposure and symptom duration of at least 6 months.

**Methods:**

Serological testing for *Bartonella henselae* and *Bartonella quintana* was performed in 224 patients. Symptoms, tick exposure, evidence of co-infection and previous treatments were evaluated. Seropositive patients were compared to a matched group (twofold larger and negative serology) from the same study cohort.

**Results:**

Seroprevalence was 7% for *B. henselae* and 1% for *B. quintana*, with one patient testing positive to both agents. Tick bites were reported by 63% of the patients in the seropositive group and 88% in the seronegative group and presumed tick exposure was more common in the seronegative group. Animal contact was equally common in both groups, along with reported symptoms. The most common symptoms were fatigue, muscular symptoms, arthralgia and cognitive symptoms. Exposure to co-infections was evenly distributed in the seropositive and seronegative groups.

**Conclusions:**

Antibodies to *Bartonella* were more common in this cohort of patients than in cohorts of healthy Swedish blood donors in previous studies but lower than those in blood donors from southern Europe. Positive *Bartonella* serology was not linked to any specific symptom, nor to (suspected) tick-bite exposure.

**Graphical abstract:**

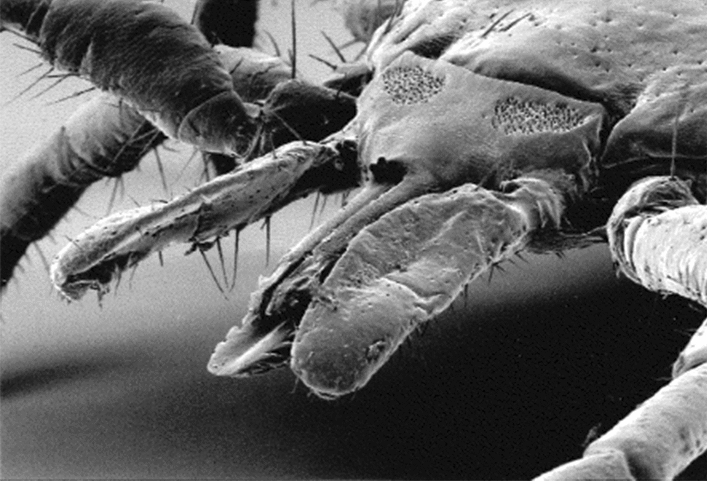

## Background

*Bartonella* spp. are slow-growing facultative intracellular bacteria that may cause various diseases, both in humans and animals. These zoonotic bacteria can be transmitted directly from animals to humans (e.g. *Bartonella henselae*) or by different blood-sucking vectors, including lice and flies. More than 40 *Bartonella* spp. are known at the present time, among which at least 14 are considered to be human pathogens [1, 2]. Most infections in humans are caused by *Bartonella quintana*, *B. henselae* and *Bartonella bacilliformis* [2], with *B. bacilliformis* being endemic to the Andes mountains of South America.

The *Bartonella* bacterium may invade and persist in red blood cells and endothelial cells and then be transferred by different arthropod vectors [3]. Ticks have been proposed, but not confirmed, as a vector for* Bartonella* transmission [4–6]. Different *Bartonella* spp. have been demonstrated in *Ixodes ricinus* ticks [7, 8], which is the tick species most often affecting humans and other large- and medium-sized animals in Sweden and Europe [9]. This tick is also the vector for *Borrelia burgdorferi* [9, 10], and co-infections in ticks have been demonstrated, a phenomenon that may result in the transmission of more than one pathogen during a tick bite [5, 11, 12]. *Bartonella henselae* and other *Bartonella* spp. have been demonstrated in the blood of patients exposed to ticks [13].

Human bartonellosis can present with a wide range of symptoms and diseases [14–17]. For example, *B. quintana* is the cause of trench fever with recurring fever, headache and bone pain [18], and *B. henselae* may cause cat-scratch disease with lymphadenopathy, fever and myalgia [19]. Atypical presentations of cat-scratch disease have also been reported with neurological and/or rheumatological symptoms [19–21]. Both *B. quintana* and *B. henselae* may cause endocarditis. Other *Bartonella* spp. have also been reported as the cause of endocarditis in a few patients [4]. Moreover, asymptomatic bacteremia with *Bartonella* spp. has been demonstrated in humans [22] and animals [7].

Patients suffering from persistent unexplained symptoms sometimes attribute these to a previous tick bite [23]. If *Bartonella* spp. are transmitted by ticks, an investigation for *Bartonella* infection may be relevant in this patient population. Therefore, we examined a cohort of Swedish patients with suspected previous tick exposure for the prevalence of antibodies against *B. henselae* and *B. quintana* and analyzed their epidemiological, clinical and baseline demographic data compared to a group of seronegative patients attending the same clinic.

## Methods

### Study population

We purposely selected participants from an exploratory study of human tick-borne infections conducted at the Center for Vector-borne Infections (CVI), Uppsala University Hospital, Uppsala, Sweden between October 2015 and December 2018 [24]. A total of 224 patients were enrolled in the principal study during this period. All patients completed standardized questionnaires on their symptoms and tick exposure and underwent a standardized medical and laboratory examination at the outpatient clinic. Patients had to fulfill at least four of seven predefined inclusion criteria of which symptom duration of > 6 months was mandatory. The other criteria were age ≥ 18 years; suspected tick-borne infection based on previous tick exposure; symptoms; laboratory findings; previous treatment for tick-borne infection; and/or suspicion of co-infection with other tick-borne infections. A summary of data on these patients has previously been published [24].

### *Bartonella* serology

Patient sera were analyzed as part of routine diagnostics at the Uppsala University Hospital, Uppsala, Sweden for IgG antibodies against *B. henselae* and *B. quintana* by indirect immunofluorescence assay (IFA) using the Anti-*Bartonella henselae*/*quintana* IIFT Mosaic kit from Euroimmun AG (Lübeck, Germany) according to the manufacturer’s instructions. Titers at 1:64 were considered to be the limit value for *B. henselae* and *B. quintana* and titers at 1:128 or higher were considered to indicate seropositivity. Positive samples were titrated to end titer.

### Microbiological testing

All patients were also examined for other tick-borne infections known to be present in Sweden, such as serological testing for *Borrelia burgdorferi*, *Borrelia afzelii* and *Borrelia garinii* (Euroimmun®, Lübeck, Germany) and *Anaplasma phagocytophilum* (Focus Diagnostics®, Cypress, CA, USA). Serological tests (IFA) for antibodies against *Babesia microti* and *Babesia divergens* were performed at the Public Health Agency of Sweden, Solna, Stockholm and the National Institute for Public Health and the Environment (RIVM), Bilthoven, the Netherlands. Serological testing (IFA) and PCR assays for *Rickettsia* spp. and tick-borne encephalitis virus (Immunozym FSME IgM and IgG, respectively; Progen Biotechnik GmbH, Heidelberg, Germany) and the PCR assay for *Candidatus* Neoehrlichia mikurensis in blood were performed at Sahlgrenska University Hospital, Gothenburg, Sweden as previously described [24].

### Statistical analysis

The Chi-square test and Fisher’s exact test were conducted using IBM SPSS Statistics (SPSS, IBM Corp., Armonk, NY, USA).

## Results

### Patient demographics

The study included 224 patients, of whom 16 (7%) were seropositive for antibodies against *B. quintana* or *B. henselae*, or both. An anti-*Bartonella* spp. IgG-seronegative group of 32 patients was matched for age, sex and region of residence from the same cohort of patients investigated at the CVI. The seropositive group included 10 women and six men with a mean age of 57 (range 23–77) years; the seronegative group comprised 20 women and 12 men with a mean age of 56 (range 20–80) years. Most patients in both groups (88%) were from central Sweden, with the majority (69% of all patients) from the region of Uppsala and Stockholm. The remaining patients were from the southern part of Sweden; none came from the northern region of Sweden.

### *Bartonella* serology

Sixteen patients (7%) were seropositive: 14 against *B. henselae*, one against *B. quintana* and one against both *B. henselae* and *B. quintana*. Anti-*Bartonella* IgG titers against *B. quintana* were between 1:64 and 1:512. The patient with the highest titer was also seropositive for *B. henselae* (titer: 1:64). For *B. henselae*, nine patients had a titer of 1:64; four, 1:128; one, 1:256; and one, 1:512. Seroprevalence was 7% for *B. henselae* and 1% for *B. quintana* in the investigated cohort.

### Co-infections

The search for other tick-borne pathogens revealed a possible infection with *Candidatus* Neoehrlichia mikurensis in one of the *Bartonella*-seropositive patients. Previous exposure to, but no ongoing infection of *B. burgdorferi*, *A. phagocytophilum* and *Rickettsia* spp. was demonstrated. The number of patients in each group is listed in Table 1. None of the patients had signs of previous exposure to or ongoing infection with tick-borne encephalitis virus, *B. divergens* or *B. microti*. There was no difference between the groups in the number of patients with previous exposure to tick-borne pathogens.

**Table 1:** Number of patients with previous exposure to tick-borne pathogens

Exposure to a pathogen	Patients positive for anti-*Bartonella* spp. IgG antibodies (*n* = 16)	Patients negative for anti-*Bartonella* spp. IgG antibodies (*n* = 32)
*Borrelia burgdorferi* sensu lato IgM/IgG	11 (69%)	21 (66%)
*Anaplasma phagocytophilum* IgG	2 (13%)	6 (19%)
*Rickettsia* spp. IgG	5 (31%)	8 (25%)
*Candidatus* Neoehrlichia mikurensis PCR	1 (6%)	0 (0%)

### Tick-exposure

Epidemiological factors related to tick exposure are shown in Table 2. Most patients who were either seropositive or seronegative for *Bartonella* reported tick bites approximately once per year. Although the difference was not statistically significant, the frequency of tick bites tended to be higher in seronegative patients. Furthermore, when the prevalence of tick bites was investigated in all anti-*Bartonella* spp. IgG-negative patients in the entire cohort investigated at the CVI clinic (*n* = 208), 79% reported tick bites. All participants in the present study reported that they walked in woods and fields over the years. Patients in the seronegative group reported berry picking (*P* = 0.012) and being on a boat or in the archipelago (*P* = 0.005) more frequently than patients in the seropositive group. When we looked at tick exposure only in the group of patients with a titer > 1:64 (i.e. 7 patients) and compared these patients to the seronegative group, we found was no difference in terms of the factors related to tick exposure. Travel history was not assessed due to incomplete or missing information on this variable from the patients.

**Table 2:** Epidemiological factors related to tick exposure in *Bartonella-*seropositive and -seronegative patients

Epidemiological factors	Patients positive for anti-*Bartonella* spp. IgG antibodies (*n* = 16)	Patients negative for anti-*Bartonella* spp. IgG antibodies (*n* = 32)
Walking In woods or fields	15 (94%)	32 (100%)
Gardening	10 (63%)	28 (88%)
Tick bite	10 (63%)	28 (88%)
Berry picking	5 (31%)	23 (72%)
Boat/archipelago	5 (31%)	24 (75%)
Hunting	2 (13%)	4 (13%)
Golf	1 (6%)	2 (6%)
Pet animal(s)	7 (44%)	15 (47%)
Dog(s)	5 (31%)	9 (28%)
Cat(s)	2 (13%)	8 (25%)
Horse(s)	2 (13%)	2 (6.3%)

### Symptoms

Reported symptoms in *Bartonella*-seropositive and -seronegative patients are listed in Table 3. The most frequently reported symptoms in the seropositive group were fatigue, muscular symptoms, arthralgia and sleeping problems. Muscular symptoms included muscle pain, spasms, muscle weakness and cramps, while sleeping problems included insomnia, prolonged sleep duration and daytime sleeping. In the seronegative group, fatigue was also a common symptom. Both the seropositive and negative groups rated fatigue as severe. In the seronegative group cognitive symptoms (e.g. confusion, concentration difficulties, difficulty processing new information, searching for names, forgetfulness, disorientation and dysphasia) were the most common symptoms. In three of the patients with previous Bell’s palsy, the serological testing of the cerebrospinal fluid was positive for *Borrelia*, which was interpreted as previous neuroborreliosis. There was no significant statistical difference between the two groups in terms of symptoms. Comparison of the symptoms in the group of patients with a positive anti-*Bartonella* spp. IgG titer > 1:64 (i.e. 7 patients) to those of the seronegative group revealed that cognitive symptoms were more common (*P* = 0.022) in the seronegative group. However, this finding should be considered uncertain due to the small size of the groups.

**Table 3:** Reported symptoms in *Bartonella*-seropositive and *Bartonella*-seronegative patients

Symptoms	Patients positive for anti-*Bartonella* spp. IgG antibodies (*n* = 16)	Patients negative for anti-*Bartonella* spp. IgG antibodies (*n* = 32)	Total (*n* = 48)
Fatigue	15 (94%)	27 (84%)	42 (88%)
Sleeping problems	11 (69%)	25 (78%)	36 (75%)
Headache	9 (56%)	24 (75%)	33 (69%)
Cognitive symptoms	10 (63%)	28 (88%)	38 (79%)
Dizziness	5 (31%)	19 (59%)	24 (50%)
Vertigo/motion sickness	3 (19%)	12 (38%)	15 (31%)
Falling sensation	3 (19%)	8 (25%)	11 (23%)
Stinging/burning sensation	7 (44%)	14 (44%)	21 (44%)
Previous Bell’s palsy (reported by patient)	1 (6%)	7 (22%)	8 (17%)
Contact hypersensitivity	5 (31%)	7 (22%)	12 (25%)
Sound sensitivity	6 (38%)	16 (50%)	22 (46%)
Other hearing symptoms	8 (50%)	19 (59%)	27 (56%)
Light sensitivity	5 (31%)	10 (31%)	15 (31%)
Other visual symptoms	7 (44%)	16 (50%)	23 (48%)
Psychiatric symptoms^a^	9 (56%)	24 (75%)	33 (69%)
Muscular symptoms	14 (88%)	26 (81%)	40 (83%)
Arthralgia	13 (81%)	27 (84%)	40 (83%)
Back pain	10 (63%)	20 (63%)	30 (63%)
Joint stiffness	10 (63%)	21 (66%)	31 (65%)
Neck stiffness	9 (56%)	22 (69%)	31 (65%)
Joint swelling	7 (44%)	12 (38%)	19 (40%)
Enlarged lymph nodes (reported by patient)	5 (31%)	6 (19%)	11 (23%)
Fever (reported by patient)	2 (13%)	13 (41%)	15 (31%)

^a^Including depression, anxiety, mood swings and psychotic symptoms

### Antibiotic treatment

Previous antibiotic treatment in *Bartonella*-seropositive and *Bartonella*-seronegative patients is shown in Table 4. Three patients had been treated with ≥ 2 different antibiotics and three patients reported ≥ 2 courses of treatment with the same sort of antibiotics. Doxycycline was the most commonly used antibiotic, reported by 38% in the seropositive and 28% in the seronegative group. All other antibiotics were reported only by very few patients. Two patients in the *Bartonella*-seropositive group were recommended doxycycline treatment; one patient was positive for *Candidatus* Neoehrlichia mikurensis in the blood and had a titer against *B. henselae* of 1:128, and the other patient had a titer of 1:256 against *B. henselae* that persisted during follow-up 2 months later. These findings together with long-lasting diffuse symptoms and no previous treatment with antibiotics effective against *Bartonella* infection resulted in the recommended treatment.

**Table 4:** Previous antibiotic treatment in *Bartonella*-seropositive and *Bartonella*-seronegative patients

Antibiotic	Patients positive for anti-*Bartonella* spp. IgG antibodies (*n* = 16)	Patients negative for anti-*Bartonella* spp. IgG antibodies (*n* = 32)
Doxycycline	6 (38%)	9 (28%)
Other macrolide	0 (0%)	1 (3%)
Phenoxymethylpenicillin	3 (19%)	1 (3%)
Beta lactam antibiotics other than phenoxymethylpenicillin	1 (6%)	1 (3%)
Clindamycin	1 (6%)	0 (0%)
Ciprofloxacin	0 (0%)	1 (3%)
Total, any antibiotic treatment	10 (63%)	11 (34%)

## Discussion

Many patients suffer from persistent medically unexplained symptoms, and if these patients have been exposed to ticks, an investigation into possible tick-borne infection may be advisable. Whether *Bartonella* infection should be included in this investigation has yet to be determined. In the present study of Swedish patients with presumed tick exposure and persistent unexplained symptoms, we demonstrated a slightly higher seroprevalence against *B. henselae* and *B. quintana* than reported in previous studies of healthy blood donors [25, 26]. However, a positive *Bartonella* serology could not be linked to any specific symptom or to a higher prevalence of any epidemiological risk factor related to tick exposure.

Ticks are considered to be one of the most important vectors in Europe, but infected patients may not always have noted a tick bite [27]. In several studies, PCR assays have demonstrated the presence of *Bartonella* spp. in ticks (including *I. ricinus*), both in Europe and the USA [1, 6, 12]. However, in Europe, the prevalence of *Bartonella* spp. in ticks varies greatly from only 0.6% in Denmark [28] to 11.8% in Germany and 38.2% in France [7]. In Sweden, the presence of *Bartonella* spp. in ticks has been investigated in two studies: one on 167 ticks [29] collected in central Sweden and the second on 1663 ticks collected in Sweden and the Åland Islands, Finland [30]. Both studies failed to demonstrate *Bartonella* spp. in the examined ticks.

The seroprevalence of antibodies against *Bartonella* spp. differs between countries. In Europe, the seroprevalence against *B. henselae* in healthy populations is higher in the central and southern countries, ranging from 19% in Germany to 23% in Austria to 57% in Croatia [31–33]. In Sweden, the seroprevalence against *B. henselae* is 3% in orienteers [25], 14% in drug addicts [34] and 29% in a homeless population [35], and ranges from 1 to 1.6% in healthy blood donors [25, 26]. A high seroprevalence against *Bartonella* spp. in populations of homeless and addicts is also known from other studies [22, 36, 37]. The present study demonstrated a seroprevalence of 7% against *B. henselae*, which is higher than that reported previously in Sweden (except for homeless persons), even in persons at high risk of exposure to tick bites (such as orienteers). The prevalence of antibodies against *B. quintana* was 1% in the current study, which is somewhat higher than previously demonstrated in healthy blood donors (0.2–0.3%) [25, 26] but lower than in values reported for orienteers (3%) and a homeless population (4.2%) [25, 35]. One patient in the present study had antibodies against both *B. henselae* and *B. quintana*, which may represent cross-reactivity; the cross-reactivity between these species has been shown in other studies to be high [38, 39]. Unfortunately, information on travel history was missing for many patients so no conclusions could be made on the role of travel on the seroprevalence against *Bartonella* spp.

The patients included in the present study were all being investigated for possible tick-borne infections. Hence, all had most likely been exposed to ticks to some extent. However, our investigation of the different epidemiological factors associated with tick exposure did not show a higher prevalence of any factor in patients with positive *Bartonella* serology. Rather, there was a tendency to a higher prevalence of some factors in the group with negative serology; these included, in particular, a higher frequency of gardening, berry picking in forested areas and being on a boat in the archipelago. Also, previous tick bites tended to be more common in the seronegative group, as did possession of a cat. This latter finding was against expectation, as it was expected that the seropositive group would have more contact with cats given that cat scratches are the primary mode of transmission of *B. henselae* to humans [19]. However, one previous serological study of cats in Sweden showed a seroprevalence of 1% against *B. henselae* [40], and when blood from cats was investigated for *B. henselae* with culture or PCR assays the prevalence was 2% [41].

Co-infections are known to occur both in ticks and humans, including *Bartonella* spp. [11, 42]. Most patients in our study showed signs of previous exposure to at least one other tick-borne pathogen, with *B. burgdorferi* being the most common. No difference between the two groups was noted for co-infections. As a result of the study design, a high prevalence of exposure to various tick-borne pathogens is not surprising.

Cat-scratch disease is caused by *B. henselae* and usually presents with fever and enlarged lymph nodes. However, atypical symptoms occur in up to 20% of patients, mostly in adults [19]. Neurological symptoms, such as meningoencephalitis, myelitis and neuroretinitis, have been described in up to 10% of cases [19], and musculoskeletal manifestations, including myalgia and arthralgia/arthritis, also appear in up to 10% of patients [43]. An investigation of patients examined by a rheumatologist demonstrated a high *Bartonella* seroprevalence and a high rate of positive *Bartonella* PCR in the blood [21]. The symptoms most frequently reported by patients in our study were fatigue, arthralgia, muscular symptoms, sleeping problems and cognitive symptoms. These symptoms were reported at similar frequencies from both seropositive and seronegative patients, with the exception of cognitive symptoms, which were more common in the seronegative group. In our seropositive patients, fatigue was the single most common symptom (frequency 94%).

## Conclusions

We have demonstrated a somewhat higher seroprevalence of antibodies against *B. henselae* and *B. quintana* in presumed tick-exposed patients than reported previously in blood donors in Sweden. However, *Bartonella*-seropositive patients did not have a higher prevalence of epidemiological risk factors associated with tick exposure, nor did they have more tick bites, when compared to *Bartonella*-seronegative patients from the same study cohort. Symptoms were also the same in both seropositive and seronegative patients. Accordingly, these findings do not add support to ticks being vectors for *Bartonella* spp. transmission. Why the prevalence of *Bartonella* antibodies is higher in this population remains to be determined.

## Data Availability

Raw data that are not included in the main text can be shared with researchers upon reasonable request.
